# mRNA-engineered T cells against telomerase: a novel immunotherapy approach for cancer

**DOI:** 10.17179/2025-8327

**Published:** 2025-05-28

**Authors:** Mudasir Maqbool, Zulfkar Qadrie, Amita Joshi Rana, Sumel Ashique, Md Sadique Hussain

**Affiliations:** 1 Department of Pharmacology, Government Medical College, Kanth Bagh, Baramulla 193101, Jammu and Kashmir, India; 2 College of Pharmacy, Graphic Era Hill University, Bhimtal, Uttarakhand 263136, India; 3 Department of Pharmaceutical Technology, Bharat Technology, Uluberia-711316, West Bengal, India; 4 Uttaranchal Institute of Pharmaceutical Sciences, Uttaranchal University, Prem Nagar, Dehradun 248007, Uttarakhand, India

## ⁯⁯

Cancer immunotherapy has progressed through the development of engineered T-cell approaches known as adoptive cell therapy (ACT). Standard ACT protocols using chimeric antigen receptor (CAR)-T cells along with T-cell receptor (TCR)-T cells pose major safety risks due to unwanted side effects coupled with cytokine release syndrome. To address these concerns, researchers have devised an innovative strategy involving the transient expression of TCRs via mRNA to target the nearly universal cancer antigen telomerase.

This mRNA-based telomerase-specific T-cell therapy involves modifying T lymphocytes to recognize telomerase, an enzyme that is predominant in most cancerous cells but rarely present in healthy somatic cells. This technique facilitates the accurate identification and elimination of cancerous cells by the administration of synthetic mRNA that encodes telomerase-TCRs, while sparing normal tissues. Compared to viral vector-based techniques, mRNA transfection offers a transient receptor expression, reducing the risk of long-term detrimental impacts and insertional mutagenesis (Bulcha et al., 2021[1], Gómez-Aguado et al., 2020[4]). Furthermore, the flexibility of mRNA systems enables rapid adaptation to tackle tumor heterogeneity and immune evasion (Chen et al., 2024[2]). Initial investigations have shown promising anticancer effectiveness and safety, highlighting the promise of mRNA-based telomerase-specific T-cell therapy as a versatile and non-viral approach to personalized cancer treatment (Hager et al., 2020[5]).

This approach has been validated by the successful cloning and production of telomerase-specific TCRs, followed by their transient implementation in both CD4⁺ and CD8⁺ T-cell subsets. The technique provides T-cells with accurate tumor cell detection while avoiding permanent genomic alterations associated with viral vector-based TCR methods (Kyte et al., 2019[6], Philip et al., 2014[8]). The targeting of T helper (Th) cells against telomerase leads to amplified anti-tumor immunity, allowing the immune system to overcome cancer defenses and sustain therapeutic response (Samadani et al., 2021[9]).

Radium-4, a promising TCR isolated from the blood of a pancreatic cancer patient vaccinated with the hTERT peptide 611-626, has shown notable results. Research shows that introducing Radium-4 TCR through mRNA electroporation into T-cells demonstrated effective tumor cell killing, particularly against melanoma and patient-derived ascites cells, without harming healthy tissues. Preclinical studies using mouse xenograft models revealed that Radium-4 TCR treatment resulted in tumor regression and improved survival outcomes, thus indicating its potential as a solid tumor immunotherapeutic agent (Dillard et al., 2021[3]).

Research have yielded encouraging results in Phase 1 studies evaluating the safety profile of Radium-4-based T-cell therapy in patients with metastatic non-small cell lung cancer. This strategy, which employs mRNA electroporation to activate MHC class II-restricted TCRs, supports transient antigen expression across diverse HLA backgrounds. It minimizes toxicity while preserving potent antitumor activity (Maggadóttir et al., 2022[7]). These findings provide a strong foundation for advancing the clinical development of mRNA-based telomerase-specific TCR therapies, offering a potentially safer and effective immunotherapeutic option for cancer treatment.

### Future Perspectives and Conclusion

mRNA-based telomerase-specific T-cell therapy offers a promising advancement in cancer immunotherapy by combining targeted antitumor activity with a favorable safety profile. The transient expression of TCRs via mRNA enables precise recognition of telomerase-expressing cancer cells while minimizing the risks associated with permanent genetic modification. The successful preclinical and early clinical results of Radium-4 TCR further highlight its potential as a non-viral, adaptable, and effective therapeutic strategy for solid tumors. Continued clinical development and optimization of this approach may pave the way for a new generation of personalized and safer cancer treatments.

## Declaration

### Acknowledgments

None.

### Author contributions

Mudasir Maqbool: Data curation, Writing - original draft. Zulfkar Qadrie: Data curation, Investigation, Writing - original draft. Md Sadique Hussain: Conceptualization, Software, Writing - review & editing. Amita Joshi Rana: Data Collection, Formal Analysis, Software. Sumel Ashique: Investigation, Writing - review & editing. 

All authors have approved the final version of the manuscript.

### Funding

None.

### Competing Interest 

Declared None.

### Data Availability

No data was used for the research described in the article.

### Ethical Approval

Not Applicable.

### Informed Consent

Not Applicable.

### Consent for Publication

Not Applicable.

### Clinical Trials Number

Not Applicable.

### Declaration of Generative AI and AI-assisted technologies in the writing process

During the preparation of this work, the author(s) used ChatGPT to correct the grammatical and typographical errors in the manuscript. All authors have approved the final version of the manuscript.
